# Effects of onion extracts (*Allium cepa*) inclusion in diets on growth performance, carcass characteristics, and bone morphometric of broiler chickens

**DOI:** 10.5713/ab.22.0399

**Published:** 2023-02-26

**Authors:** E. Malematja, T. G. Manyelo, J. W. Ng’ambi, M. F. D. Nemauluma, S. D. Kolobe

**Affiliations:** 1 Department of Agricultural Economics and Animal Production, University of Limpopo, Private Bag X1106, Sovenga 0727, South Africa

**Keywords:** Bone Morphology, Broiler Chickens, Carcass Characteristics, Growth Performance, Onion, Sensory Evaluation

## Abstract

**Objective:**

Animal feed companies and researchers are currently embarking on quests for feed additives that could combat the pathogens while promoting growth as well as maintaining quality products. The current study aimed to evaluate the effects of onion extracts on growth performance, carcass quality, and bone morphometrics of broiler chickens.

**Methods:**

A total number of 200 one-day-old unsexed Ross 308 broiler chicks were assigned to 5 treatment groups, replicated 4 times. Each replicate (pens) held 10 chickens in a completely randomized design. The experimental diets were then randomly allotted to the pens which act as experimental units. The isoenergetic and isonitrogenous diets were formulated by including onion extracts at 0, 5, 10, 15, or 25 g/kg in a complete broiler diet. Feed intake, and body weight, were recorded then used to calculate feed conversion ratio. At the end of the experiment (42 days), four chickens from each pen were randomly selected for slaughter for carcass, bone morphology and sensory evaluation.

**Results:**

Results showed that onion extract supplementation did not affect (p>0.05) growth performances and meat sensory evaluation. However, there was a significantly increased (p<0.05) meat shear force in groups receiving onion extracts dietary treatments. Furthermore, onion extracts improved (p<0.05) bone morphology of broiler chickens in terms of weight, diameter, calcium, and phosphorous contents.

**Conclusion:**

In conclusion, onion extracts can be safely included in a commercial broiler diet as a growth promoter without causing adverse effects on growth performance traits and carcass quality in chickens. Onion extract supplementation improved tibia bone growth and strength in broiler chickens.

## INTRODUCTION

Chicken production is practiced throughout the world; however, its success is determined by managerial factors such as plane of nutrition, ambient temperatures as well as biosecurity measures [1]. Among these factors, the plane of nutrition and ideal temperatures can be corrected through good management practices, but disease control remains the major since microorganisms causing diseases are everywhere, in feeds, water, and the environment, and hence difficult to mitigate [2]. These pathogens disturb the digestive functioning when ingested with food or water, by causing inflammation in the gut, thus disturbing nutrient digestion and absorption [3], hence reducing the growth and productivity of the chickens. The emergence of microorganisms threatens the future of the poultry industry [4], therefore, animal feed companies and researchers are currently embarked on quests for feed additives that could combat the pathogens while promoting growth as well as maintaining quality products.

Feed additives maximize nutrient exploitation by the chicken, thus enhancing growth performance and feed conversion efficiency [5]. Some attempt has been made to improve chicken performance and health condition with the feeding of phytogenic additives [1]. Medicinal herb mixture of compounds or a single species can be used to improve the performance and health conditions of chickens [1]. There is evidence that onion extracts can be used to improve the growth performance and productivity of chickens [6]. Several studies have reported positive results on the use of onions in poultry production in terms of body weight (BW) gain and feed conversion efficiency [7–9]. Onion contains numerous organic compounds, flavonoids, and phenolic acids with proven antibacterial, antioxidant and hypolipidemic efficiency which are known to promote growth in poultry [10,11]. Because of genetic improvements of chickens, dietary onion inclusion levels for optimal productivity change. However, little information has been reported concerning the recommended onion inclusion levels for better performance. It is, therefore, important to determine dietary onion extracts inclusion levels for optimal performance of broiler chickens and the study aimed to generate information on the effect of onion extracts inclusion level in the diet on growth performance, carcass characteristics, and bone morphometric o broiler chickens. Such information will help in devising strategies aimed at improving the productivity and carcass quality of the broiler chickens.

## MATERIALS AND METHODS

The experiment was conducted at the University of Limpopo Livestock Unit, in Limpopo province, South Africa. The University of Limpopo lies at latitude 27.55°S and longitude 24.77°E. The study was conducted between March and April 2021 when ambient temperatures around the study area ranged 29°C to 36°C [12]. The protocol of animal handling was approved by the University of Limpopo Animal Research Ethics Committee (ULAREC Number: AREC/07/2020: PG).

### Acquisition and preparation of onion bulb (*Allium cepa*)

Fresh onions bulbs (*Allium cepa*) were purchased from ZZ2 fresh produce markets in Mooketsi, Limpopo province, South Africa. They were then cleaned, peeled, grated into small pieces, and air-dried in a well-ventilated lab with no direct sun exposure. The dried onion pieces were pulverized into powder and stored in polythene bags prior to chemical analysis [13] and feed formulation (Table 1).

### Chemical analysis

Prior to feed formulation onion extract sample and experimental feeds were analysed following AOAC [13]. The dry matter (DM) was determined by drying the samples in the oven for 24 hours at 105°C (DM; method no 930.15). The nitrogen content of the feed was using Kjeldahl method (method no 984.13) to calculate crude protein (N×6.25) [13]. Gross energy was determined using a bomb calorimeter [13] at the University of Pretoria Animal Nutrition Laboratory. Full analyses for feeds were performed at the Pietermartizburg laboratory, Kwa-Zulu Natal, South Africa according to AOAC [13].

### Birds’ management, experimental designs, and treatments

The experiment commenced with 200-day-old unsexed Ross 308 broiler chicks which were assigned to five treatment groups, each having four replicate pens holding ten chicks in a completely randomized design. The birds were reared on floor pens (120×100×80 cm) in an environmentally controlled house for 42 days. The house temperature was kept at 32°C during the first few weeks and adjusted according to their ideal temperature as the birds grow. The chickens were offered feed and water *ad libitum* and photoperiod was 23 L:1 D. The five isoenergetic and isonitrogenous diets were formulated to met the nutrient requirements of broiler chickens as recommended by the National Research Council [14] as follows; i) OE0 = Basal diets with no onion; ii) OE5 = Basal diet in which 5 g/kg of onion extracts were included; iii) OE10 = Basal diet in which 10 g/kg of onion extract were included; iv) OE15 = Basal diet in which 15 g/kg of onion extracts were included; or v) OE25 = Basal diet in which 25 g/kg of onion extract were included (Tables 2 and 3).

### Data collection

#### Growth performance parameters

The initial live weight (LW) of each chick was weighed and recorded at the start of the experiment, thereafter on weekly basis using an electronic weighing balance (Model AFP 110L). The determined LWs were used to calculate the total weight gain (TWG) and daily weight gain (DWG) of the chickens. The weighed feeds were offered in the morning and refusal were collected the following day at the same time. Thereafter, the daily feed intake (FI) was calculated by subtracting the feed offered from the feed refusal. The feed conversion ratio (FCR) was calculated as the total amount of feed consumed divided by the weight gain of a live chicken (put reference)

#### Carcass characteristics and sensory evaluation

At the age of 42 days, four chickens per replicate were randomly selected for carcass evaluation and bone morphology. Each chicken was weighed and humanely slaughtered according to the rules and regulations of the University of Limpopo Animal Research Ethics Committee. They were then hanged upside down to completely bleed out, then de-feathered, eviscerated, and finally weighed and recorded as hot carcass weight. The weight of carcasses and meat organs was determined immediately after slaughtering using an electronic weighing balance and expressed in grams as hot carcass weight and stored in cold freeze (−4). The following day, meat pH from breast-meat, thigh, drumstick, and wing at three different place was taken as ultimate pH (pHu) using a pH meter (Model 4 Corning Glass Works, Medfield, MA, USA) calibrated at pH of 7.0.

#### Shear force determination

Frozen samples of chicken breast meat were thawed for 24 h at 2°C, tagged, and boiled on an electrical stove which was set at 35°C and finished at 70°C. Cooked meat samples were cooled down to room temperature (18°C) for at least 2 hours before Warner-Bratzler shear force (WBSF) measurements as described by Dawson et al [15]. Three cylindrical samples (12.5 mm core diameter) of each cut were cored parallel to the grain of the meat and sheared perpendicular to the fibre direction using a WBSF device mounted on a Universal Instron Apparatus (crosshead speed = 200 mm/min, one shear in the centre of each core). The reported value in N represents the average of three peak force measurements of each sample.

#### Sensory evaluation

Meat samples were frozen at −40°C for 3 days and they were then thawed for 7 hours at room temperature before cooking [16]. The breast meat was prepared, the skin was left on the meat samples, and nothing was added to the meat samples to enhance the flavour of the meat. The meat samples were covered with aluminium foil to prevent water loss and placed into an oven and cooked at 105°C for approximately an hour [16]. After cooking, meat samples were cut into small 5 cm cubic pieces and served immediately. The meat was evaluated for tenderness, juiciness, and flavour. The sensory panel consisted of 20 trained panelists to rank each part on a 5-point ranking scale (Table 4). Each panelist was offered to drink lemon juice after tasting meat from each treatment before proceeding to the next treatment to wash out the previous treatment to avoid confusion of tastes.

#### Bone morphology

Right tibia bones were excised and defleshed without boiling [13]. Thereafter, bones were analysed for physical bone characteristics (weight, length, and diameter) using an electronic weighing balance and a measuring caliper. Thereafter, bone samples were analysed using AOAC (2005); ash (method 942.05). The samples were ashed in a muffle furnace at 550°C for 5 hours. Approximately 1 g of each bone ash sample was then dissolved in 10 mL of 3 M hydrochloric acid and boiled for 10 minutes. The samples were allowed to cool and filtered into a 100 mL volumetric flask. Thereafter, the volume was topped to 100 mL with deionised water and analysed for minerals [13]. Calcium content was analysed as described in AOAC [13] (method 978.02) and phosphorus (method 924.05).

### Statistical analyses

Data collected were subjected to analysis of variance using SAS version 9.3.1 software program one-way ANOVA [17], with onion inclusion extracts level as the main effect. Where there were significant differences (p<0.05) between the treatments, Tukey’s honestly significant difference test (HSD) was used for mean separation. The model y_ij_= μ+T_i_+e_ij_ was applied, where y_ij_ = response variables; μ = population mean; T_i_ = fixed effect of the ith treatment level (i = 0, 5, 10, 15, and 25 g/kg of feed) and e_ij_ = random residual error.

## RESULTS

### Growth performance of broiler chickens aged one to 42 days

Results of the effects of onion extracts inclusion level in a diet on FI, FCR, TWG, and DWG of broiler chickens aged one to 42 days are presented in Table 5. The present study showed that onion extracts inclusion levels in diets have no effects (p>0.05) FI, FCR, LW, and growth rate of broiler chickens throughout the experimental period.

### Carcass weight, organ weights, and meat pH

The study revealed that onion extracts inclusion levels used in this experiment did not affect (p>0.05) carcass, breast meat, thigh, drumstick, and wing weights in broiler chickens at slaughter age (Table 6). Similarly to previous statement, onion extracts inclusion levels in diets did not affect (p>0.05) breast meat, thigh, drumstick, and wing pH of broiler chickens at slaughter age (Table 6).

### Sensory evaluation and meat shear-force

Results of the effects of onion extracts inclusion levels in diets on meat juiciness, flavour, tenderness, and shear force (N) of broiler chickens aged 42 days are presented in Table 7. The current study indicates that adding onion extracts to broiler’s diet did not affect (p>0.05) meat juiciness, flavour, and tenderness of the chickens at slaughter age. However, onion extracts inclusion levels in diets affected (p<0.05) meat shear-force of the chickens aged 42 days.

### Bone morphology

Investigation on the effects of feeding chickens diets containing different levels of onion extracts showed that onion extracts have no effect (p>0.05) on the lengths of right tibia bones of broiler chickens aged 42 days (Table 8). However, different levels of onion extracts in diets affected (p<0.05) weight and diameter, calcium, and phosphorus contents of right tibia bones of the chickens aged 42 days.

## DISCUSSION

Despite the present of phytochemical compounds in onion bulbs which could reduce harmful microbial population in the gastrointestinal tract and enhance gut health, nutrient digestion, absorption, and promote growth in chickens [18], the current study indicated that onion extracts inclusion in diets have no effect on FI, FCR, TWG, and DWG of broiler chickens aged one to 42 days.

The results of the current study are in line with the findings made by An et al [19] who reported that adding different levels of onion extracts did not affect FI and FCR in Mini-white broiler chickens. Similarly, Farahani et al [20] observed no effect on FI, FCR, and GR of Ross broiler chickens fed basal diet containg 10 g/kg onion extracts. This suggests that adding onion extracts in diets did not alter the physiochemical parameters of the diet and thus did not affect its functional properties. Conversely, Goodarzi et al [1] and Al-Ramamneh [21] investigated the effect of using onion extracts in diets and observed improved FI, FCR, and BW of broiler chickens throughout the growing period. Similar results have been reported by Tashla et al [18], Aji et al [22], Aditya et al [23], Goodarzi et al [24], and Omar et al [25] who reported increased FI, BW gain, and LW of broiler chickens. In this study, it is clear that onion extracts can be safely included in broiler chicken’s diets at levels up to 25 g/kg of feed without causing adverse effects on growth performance of broiler chickens.

Carcass assessment is the most important aspect of broiler chicken production because it reflects the amount of meat produced. Allicin is the key active compound in onion bulbs which reduce several volatile organosurlphure compounds [6,10,11]. Also, it possesses antioxidant compounds such as flavonoid and sulfure-containing compounds which help to reduce cholesterol hence improving meat quality in terms of flavor, color, tenderness, overall acceptibility, and storage [1,6,10]. In the current study, there were no effects on carcass, thigh, and wing weights of broiler chickens at slaughter age. These findings are in line with observations made by several authors who reported no improvements in carcass and edible parts characteristics of broiler chickens fed diets containing onion extracts or extracts [1,19,22,25]. Comparing the results of the current study with the previous studies, it can be suggested that onion extracts is a suitable herbal plant to use in poultry feeds without compromising carcass yield and the sensory attributes of the meat. In contrast, Sadeli et al [26] reported that adding onion extracts or powder into chicken diets increased the slaughter and carcass weight of broiler chickens. Farahani et al [20] also assessed the effect of supplementing 10 g/kg aqueous onion extracts in Ross and Cobb broiler chickens and reported heavier breast meat in both strains. Similarly, to previous statements, adding 1 g/kg dry onion into broiler chicken diets resulted in increased carcass weight of broiler chickens aged 6 weeks [27].

Meat pH is used to measure the rate at which glycogen is converted to lactic acid after slaughtering [28]. The results of the present study indicated that onion extracts inclusion levels in diets did not affect meat ultimate pH values of broiler chickens aged 42 days and it was observed to be within the normal range (5.5 to 6.5) for poultry [29]. These findings are similar to those of Jang et al [30], who reported that dietary quercetin and methoxylated quercetin from onion did not influence meat pH in chickens after slaughter. This implies that the use of onion extracts in chicken diets does not affect glycogen levels and hence thus meat pH. However, information on the effect of onion inclusion in the diet on broiler chicken meat pH values is limited. There is, therefore, a need to do more studies to ascertain the present findings.

Onion extracts inclusion levels in diets used in the present study did not affect meat juiciness, flavour, and tenderness of broiler chickens aged 42 days. Although meat shear force was affected, obtained shear force values fall within the normal range. Factors such as handling stress, scalding temperature, and rate of rigor development could be the cause of increased meat shear force. The results of the current study are contrary to those of Ali and Zahran [31] who reported a significant decrease in meat shear force in the samples from chickens fed diets supplemented with 2 g/kg onion and garlic in drinking water compared with the control. However, there is less information reported on the effect of onion extracts inclusion in broiler diets on meat sensory attributes; therefore, more studies are recommended to verify these findings.

At 42 days of age, the results of the current study showed that there was a significant increase in weight, diameter, calcium, and phosphorous contents of right tibia bone of broiler chickens. These improvements could be due to the reason that onion bulbs are rich in minerals such as calcium and phosphorous [6]. Furthermore, onion bulbs contains idione and sulfur salts which reduce the occurrence of rheumatism [32] thus benefit in the development of skeletal system. It is clearly that the use of onion extracts at levels of 5 to 25 g/kg in diets can support tibia bone growth and strength in broiler chickens. Unfortunately, no information on the effect of onion extracts inclusion in a diet on broiler chicken bone morphology was found. Further studies are recommended to ascertain these results.

## CONCLUSION

Based on the obtained results, the present study indicates that onion extracts exhibited no effect on growth performance, carcass characteristics of broiler chickens, hence it could be used as an alternative growth promoter in poultry production without causing adverse effects on growth performance and carcass quality. In addition, dietary treatment improved bone morphology of Ross 308 broiler chickens, However, further studies on the effects of onion bulbs in chicken diets are recommended.

## Figures and Tables

**Table 1:** Proximate analysis of onion extracts

Nutrient	Composition
Moisture (%)	89.20
Crude protein (%)	1.47
Lipids (%)	0.19
Ash (%)	0.48
Carbohydrates (%)	7.35
Crude fiber (%)	1.84
Energy (kcal)	40.41

**Table 2:** Experimental diet composition for stater diet

Items	Onion extracts inclusion level (g/kg)				

	0	5	10	15	25
Feed ingredient					
Maize meal (%)	47.80	46.20	47.30	46.80	46.00
Wheat offal (%)	8.00	8.00	8.00	8.00	7.80
Full fat soya (%)	32.00	32.00	32.00	32.00	32.00
Fish extracts (%)	5.00	5.00	5.00	5.00	5.00
Limestone (%)	1.00	1.00	1.00	1.00	1.00
Bone extracts (%)	4.95	4.45	4.45	4.45	4.45
Salt (%)	0.25	0.25	0.25	0.25	0.25
Vitamin-mineral premix^1)^	0.50	0.50	0.50	0.50	0.50
Dl-Methionine (%)	0.25	0.25	0.25	0.25	0.25
L-Lysine (%)	0.25	0.25	0.25	0.25	0.25
Onion extracts(%)	0.00	0.50	1.00	1.50	2.50
Total	100.00	100.00	100.00	100.00	100.00
Calculated analysis					
CP (%)	23.00	23.00	23.00	23.00	23.00
Metabolizable energy (MJ/kg )	12.55	12.55	12.55	12.55	12.55
EE (%)	6.23	6.23	623	6.23	6.23
CF (%)	3.90	4.06	3.99	3.99	3.92
Ca (%)	1.40	1.3	1.23	1.27	1.27
Available P (%)	0.74	0.68	0.68	0.68	0.68
Lysine (%)	1.37	1.37	1.37	1.37	1.37
Methionine+cysteine (%)	0.66	0.66	0.66	0.66	0.65

CP, crude protein; EE, ether extracts; CF, crude fibre.

1) Vitamin-minerals were as follows (per kg of diet): vitamin A 12,000 IU, vitamin D_3_ 3,500 IU, vitamin E 30.0 mg, vitamin K_3_ 2.0 mg, thiamine 2 mg, riboflavin 6 mg, pyridoxine 5 mg, vitamin B_12_ 0.02 mg, niacin 50 mg, pantothenate 12 mg, biotin 0.01 mg, folic acid 2 mg, Fe 60 mg, Zn 60 mg, Mn 80 mg, Cu 8 mg, Se 0.1 mg, Mo 1 mg, Co 0.3 mg, I 1 mg.

**Table 3:** Ingredients and nutrient composition of the experimental diets (Grower diet)

Items	Onion extracts inclusion level (g/kg)				

	0	5	10	15	25
Feed ingredient					
Maize (%)	51.00	48.00	48.00	47.50	47.00
Wheat offal (%)	12.00	13.00	12.50	12.50	12.00
Full fat soya (%)	28.00	30.00	30.00	30.00	30.00
Fish extracts (%)	4.00	4.00	4.00	4.00	4.00
Limestone (%)	1.00	1.00	1.00	1.00	1.00
Bone extracts (%)	2.95	2.45	2.45	2.45	2.45
Salt (%)	0.25	0.25	0.25	0.25	0.25
Vitamin-mineral premix^1)^	0.25	0.25	0.25	0.25	0.25
Dl-Methionine (%)	0.25	0.25	0.25	0.25	0.25
L-Lysine (%)	0.25	0.25	0.25	0.25	0.25
Onion extracts (%)	0.00	0.50	1.00	1.50	2.50
Coccidiostat (%)	0.05	0.05	0.05	0.05	0.05
Total	100.00	100.00	100.00	100.00	100.00
Calculated analysis					
CP (%)	20.01	20.02	20.02	20.02	20.01
Metabolizable energy (MJ/kg)	16.80	16.80	16.80	16.80	16.80
EE (%)	6.23	6.23	623	6.23	6.23
CF (%)	3.90	4.06	3.99	3.99	3.92
Ca (%)	1.40	1.3	1.23	1.27	1.27
Available P (%)	0.74	0.68	0.68	0.68	0.68
Lysine (%)	1.37	1.37	1.37	1.37	1.37
Methionine+cysteine (%)	0.66	0.66	0.66	0.66	0.65

CP, crude protein; EE, ether extracts; CF, crude fibre.

1) Vitamin-minerals were as follows (per kg of diet): vitamin A 12,000 IU, vitamin D_3_ 3,500 IU, vitamin E 30.0 mg, vitamin K_3_ 2.0 mg, thiamine 2 mg, riboflavin 6 mg, pyridoxine 5 mg, vitamin B_12_ 0.02 mg, niacin 50 mg, pantothenate 12 mg, biotin 0.01 mg, folic acid 2 mg, Fe 60 mg, Zn 60 mg, Mn 80 mg, Cu 8 mg, Se 0.1 mg, Mo 1 mg, Co 0.3 mg, I 1 mg.

**Table 4:** Evaluation score used by panelists

Score	Meat		

	Flavour	Tenderness	Juiciness
1	Very bad flavour	Too tough	Extremely dry
2	Poor flavour	Tough	Dry
3	Neither bad nor good flavour	Neither tough nor tender	Neither dry nor juicy
4	Good flavour	Tender	juicy
5	Very good flavour	Too tender	Too juicy

**Table 5:** Effect of onion extracts inclusion in a diet on feed intake, feed conversion ratio, total weight gain, and daily weight gain of Ross 308 chickens aged one to 42 days

Parameter	Onion extracts inclusion level (g/kg DM)^1)^				

	0	5	10	15	25
Feed intake (g/broiler/d)	193.53±6.993	196.79±5.484	202.94±6.223	196.70±7.654	199.59±6.142
Feed conversion ratio	2.26±0.046	2.16±0.069	2.15±0.050	2.11±0.096	2.23±0.058
Total weight again (g/broiler aged 21 d)	2,930.38±45.585	2,993.63±29.750	3,000.96±82.456	2,997.88±159.819	2,990.50±74.389
Daily weight gain (g/broiler/d)	85.60±4.362	90.99±0.853	93.10±4.493	90.37±5.857	89.26±2.945

Values presented as mean±standard error.

1) Treatments were onion inclusion level in the diet at 0, 5, 10, 15, or 25 g/kg.

**Table 6:** Effect of onion extracts inclusion on meat pH, carcass and carcass part weights (g) of broiler chickens at slaughter age

Variables	Onion extracts inclusion level (g/kg DM)^1)^				

	0	5	10	15	25
Carcass weight	2,378.58±67.432	2,380.92±8.584	2,423±59.399	2,379.42±88.336	2,379.08±81.699
Meat parts weight					
Breast meat	939.85±24.844	942.00±18.399	952.21±7.469	941.60±24.383	940.40±20.892
Thigh	141.66±9.454	144.03±3.829	146.66±4.910	143.16±4.512	143.50±6.959
Drumstick	155.06±4.312	158.46±6.163	159.56±3.1068	156.93±5.368	156.83±2.471
Wing	107.30±5.846	109.25±2.183	111.06±5.875	110.01±2.934	110.55±2.450
Meat pH					
Breast-meat	5.61^a^±0.064	5.60^a^±0.081	5.60^a^±0.050	5.60^a^±0.004	5.60^b^±0.077
Thigh	6.12±0.151	5.93±0.190	6.12±0.117	6.09±0.084	6.20±0.045
Drumstick	6.26±0.078	6.28±0.065	6.15±0.050	6.13±0.025	6.13±0.132
Wing	6.14±0.012	5.99±0.027	5.84±0.060	5.90±0.127	6.01±0.091

Values presented as mean±standard error.

DM, dry matter.

1) Treatments were onion inclusion levels in the diet at 0, 5, 10, 15, or 25 g/kg DM.

a,b Means with different superscripts in the same row indicate significant differences between treatments (p<0.05).

**Table 7:** Effect of onion extracts inclusion levels in diets on meat juiciness, flavour, tenderness, and shear-force of broiler chickens aged 42 days

Parameters	Onion extracts inclusion level (g/kg DM)^1)^				

	0	5	10	15	25
Juiciness	4.82±0.139	4.81±0.220	4.80±0.289	4.80±0.176	4.80±0.333
Flavour	3.83±0.927	3.43±0.338	3.12±0.147	4.07±0.080	4.10±0.416
Tenderness	4.40±0.305	4.03±0.284	4.00±0.583	4.06±0.581	3.38±0.310
Shear force	7.53^b^±0.152	9.87^a^±0.816	6.20^b^±0.317	7.55^b^±0.475	6.67^b^±0.232

Values presented as mean±standard error.

DM, dry matter.

1) Treatments were onion inclusion level in the diet at 0, 5, 10, 15, or 25 g/kg DM.

a,b Means with different superscripts in the same row indicate significant differences between treatments (p<0.05).

**Table 8:** Effect of onion extracts inclusion levels in diets on weight (g), length (mm) and diameter (mm), and calcium and phosphorus (%) contents of right tibia bones of Ross 308 broiler chickens aged 42 days

Parameters	Onion extracts inclusion level (g/kg DM)^1)^				

	0	5	10	15	25
Weight	15.98±0.480	16±0.652	16.10±0.332	16.13±0.344	16.15^a^±0.104
Length	98.67±0.600	98.66±0.875	99.33±0.766	98.66±0.833	99±0.82
Diameter	8.66^b^±0.440	9.16^ab^±0.166	9.33^ab^±0.440	8.83^ab^±0.166	9.83^a^±0.440
Calcium	30.88^bc^+0.483	31.22^ab^+0.526	31.72^ab^+0.386	33.72^a^+0.637	32.06^ab^+1.030
Phosphorous	10.15^b^+0.078	9.02^c^+0.017	8.59^d^+0.101	10.36^b^+0.098	11.49^a^+0.063

Values presented as mean±standard error.

DM, dry matter.

1) Treatments were onion inclusion level in the diet at 0, 5, 10, 15, or 25 g/kg DM.

a–d Means with different superscripts in the same row indicate significant differences between treatments (p<0.05).
